# Medial malleolar window approach for varus-type tibial pilon fractures: a retrospective study

**DOI:** 10.1186/s12891-023-06444-4

**Published:** 2023-05-06

**Authors:** Kangyong Yang, Guodong Shen, Qian Zheng, Haiyun Yang, Hongning Zhang, Xue Li, Yanqing Tan, Yongzhan Zhu

**Affiliations:** 1 The Eighth School of Clinical Medicine, Guangzhou University of Chinese Medicine, Foshan, Guangdong China; 2 Department of Foot and Ankle Orthopedics, Foshan Hospital of Traditional Chinese Medicine, Foshan, Guangdong 528000 China

**Keywords:** Medial malleolar, Surgical approach, Varus, Pilon fracture

## Abstract

**Purpose:**

Choosing a suitable surgical approach is crucial and challenging for type C pilon fractures. This article aims to explore the clinical efficacy of the medial malleolar window approach for varus-type tibial pilon fractures.

**Methods:**

A retrospective analysis was conducted on 38 patients with type C varus-type pilon fractures treated between May 2018 and June 2021. In total, 16 cases underwent surgical treatment through the medial malleolar window approach and 22 cases were treated with the traditional anteromedial approach combined with a posterior approach. The operation time, hospitalization time, fracture healing time, the American Orthopedic Foot and Ankle score, Visual Analogue Scale, and complications were recorded to comprehensively evaluate the clinical efficacy of the technique. Fracture reduction quality was evaluated using the criteria proposed by Burwell and Charnley.

**Results:**

All patients were followed up. No patients presented delayed union or nonunion. Compared with the conventional approach, the medial malleolar window approach had the advantage of better clinical effect recovery and better fracture reduction (P < 0.05). Meanwhile, the medial malleolar window approach had a shorter operation time, although the statistics suggest no significant difference with the control group. No implant exposure or infection occurred. There was good wound healing at two weeks after surgery in all but two cases. Local wound edge necrosis developed in one case in the medial malleolar window approach group, and the wound could not be closed at one stage in another case in the conventional group because of excessive tension, requiring secondary closure.

**Conclusion:**

The medial malleolar window approach provides excellent exposure to type C pilon fractures, allowing for satisfactory fracture reduction and functional rehabilitation. The medial window approach is recommended for varus-type pilon fractures, which can effectively avoid a posterior incision and reduce the operation time.

## Introduction

Tibial pilon fractures account for about 1% of lower extremity fractures and 5–7% of tibial fractures [1, 2]. They are usually caused by an axial compressive force on the tibial leading to multiple fragments, compression of the articular surface, and abnormal lower limb alignment. Despite the relative rarity of this type of fracture, it is important to note that pilon fractures have become more complex and intractable, and complex pilon fractures (AO/ OTA43C type) account for 30% of fractures caused by high-energy trauma [1].

Complex pilon fractures are often accompanied by severe soft tissue injury with severe soft tissue injuries, including swelling of the ipsilateral limb and tension blisters or bloody blisters. Even after detumescence, one-stage traction, or external fixation, delayed surgery still carries the risk of infection at the incision, necrosis, fixation failure, or even amputation [3].

Given the complications of complex pilon fractures, there is controversy in clinical practice regarding complex pilon fractures, including the choice of internal fixation and treatment strategy. To reduce the risk of complications, various techniques have been proposed for the management of complex pilon fractures. While staged surgical strategy with one-stage external stent fixation used to be advocated [4], minimally invasive surgeries and related techniques [5–8], are now more common strategies.

The choice of surgical approach is also controversial. Multiple surgical approaches have been introduced for pilon fractures, including the lateral, anterolateral, anteromedial, posteromedial, and posterolateral approaches [9], to name a few. Double incisions are still preferred by most orthopedists, but this poses the risk of complications such as skin necrosis and incision closure difficulties [10]. Therefore, orthopedic surgeons strive for a better surgical approach. Several scholars [11–14] have recently proposed new surgical approaches, including some that are completely novel and have not been introduced in the past and others that are improvements of traditional surgical approaches. These new approaches aim to achieve effective fracture reduction and internal fixation and reduce postoperative complications.

A standard medial approach is a common approach for ankle fractures or cases that need medial malleolus osteotomy [15, 16]. However, for pilon fractures, the medial approach is often chosen as it is a minimally invasive approach and does not make a direct open incision [9]. For the medial approach, after the medial column of the distal tibia has been reduced, a plate is inserted through the small incision at the tip of the medial malleolus followed by the distal and proximal screw fixation, which avoids exposing the metaphysis to prevent interference with the vasculature of the soft tissues and bones [9]. We wondered whether a conventional medial approach would be appropriate for pilon fractures that present with a large medial malleolar fragment since this approach theoretically allows for direct visualization of the distal tibial articular components by inverting the medial malleolar fragment. In practice, we were surprised to find that the distal tibial surface can be exposed by inverting the medial malleolar fragment through the standard medial approach, and there is adequate visualization allowing for good reduction and internal fixation of the fracture in varus-type tibial pilon fractures. To our knowledge, this approach for pilon fractures has not been reported yet in clinical practice, so we have named this technique the medial malleolar window approach and it is characterized by an inversion of the medial malleolar fragment through a standard medial approach.

This study aims to introduce the medial malleolar window approach for the treatment of type C pilon fractures for the first time. We anticipate that this approach will improve the surgical treatment of pilon fractures.

## Methods

### Patient population

This is a retrospective case-control study and this study was approved by the Ethics Committee of Foshan Hospital of TCM and all participants provided written informed consent. Between May 2018 and June 2021, a total of 38 patients who sustained pilon fractures (AO/OTA43-C) [17] and presented to the Department of Foot and Ankle Surgery were enrolled in this study. Of these,16 patients underwent surgical treatment through the medial malleolar window approach while the remaining 22 patients chose the traditional anteromedial approach or extensile approach, combing a posterior approach.

Patients were eligible for inclusion if they had recent varus-type tibial pilon fractures based on ankle position at the time of injury. Patients were excluded if they had the following conditions: other types of pilon fractures, such as valgus-type tibial pilon fractures, neutral-type tibial pilon fractures, etc.; open fractures; pathological fractures; the presence of severe associated systemic diseases; the presence of compartment syndrome.

Age, sex, injured side, Tscherne soft tissue classification, prognostic risk factors, follow-up time, hospitalization time, time to operative treatment, operation time, and fracture healing time were recorded.

The quality of postoperative fracture reduction was categorized as anatomic, fair, or poor according to the Burwell-Charnley criteria [18]. All patients were assigned an American Orthopedic Foot and Ankle Society Score (AOFAS) at the last follow-up, and the clinical outcome was categorized as excellent, fair, or poor. Visual Analogue Scale (VAS) was checked at the last follow-up. Complications were recorded for all participants. Using this information, the clinical efficacy of this approach was analyzed.

The patients were usually followed up at 2 weeks, 1 month, 3 months, 4 months, 6 months, 12 months, and 18 months after surgery in the clinic. Emphasis was placed on observing the wound healing at the first two follow-ups, and the functional rehabilitation and the observation of fracture healing were the key points at the 3rd and 4th follow-ups, and the last follow-up was focused on assessing the functional recovery, with recording the AOFAS and VAS scores.

### Surgical technique

#### Medial malleolar window approach group

**Fig. 1:**
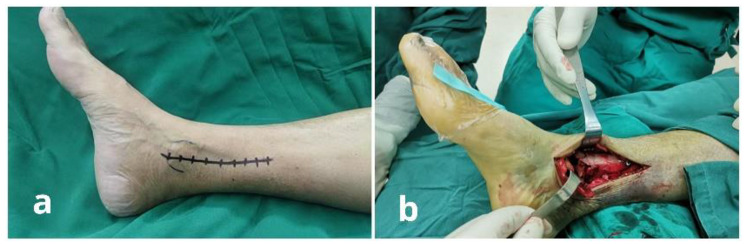
(**a**) Photograph of the standard medial incision: the incision centered over the medial malleolus was made starting 1 cm below the tip of the medial malleolus to the proximal 8–10 cm of the ankle joint. (**b**) The medial malleolar window appeared after the medial malleolar fragment was reflected plantarward and retracted inferiorly

The patients were positioned supine under spinal anesthesia and a peripheral nerve block with a tourniquet on the base of the ipsilateral thigh. Antibiotics were administered 30 min before the surgery and the tourniquet was inflated to 300 mm Hg. An incision was made directly over the medial malleolus, 1 cm below the tip of the medial malleolus, and extending proximally about 8–10 cm in length or further if necessary (Fig. 1a). Care was taken to protect and preserve the great saphenous vein and saphenous nerve when incising the subcutaneous tissue. The anterior side of the ankle joint was exposed along the anterior edge of the medial malleolus, and then the tibial tendon sheath was cut along the posterior edge of the medial malleolus. After confirming that the soft tissue around the medial malleolus had been completely released, the medial malleolar fragment was slowly moved plantarward (Fig. 1b), and then the medial malleolar fragment attached to the deltoid ligament was retracted inferiorly with a towel clamp. A stripper was subsequently used to dissect the metaphyseal periosteum.

Once the medial malleolar fragment was inverted, the articular surface of the distal tibia and the talus dome could be seen clearly. To get better exposure to the tibiotalar joint and facilitate operative manipulation, a bump was placed under the ipsilateral ankle to move the ankle into a valgus position. Removing the hematoma and debris was beneficial for improving the visualization but caution should be taken to preserve the articular fragments during this process. As the soft tissues of the anterior fragments and the posterior fragments were dissected, each displaced fragment of the distal tibial articular surface and the exact location of the die-punch segments could be visualized. It should be noted that the posterior neurovascular bundle should be carefully protected when dissecting the posterior fragments.

**Fig. 2:**
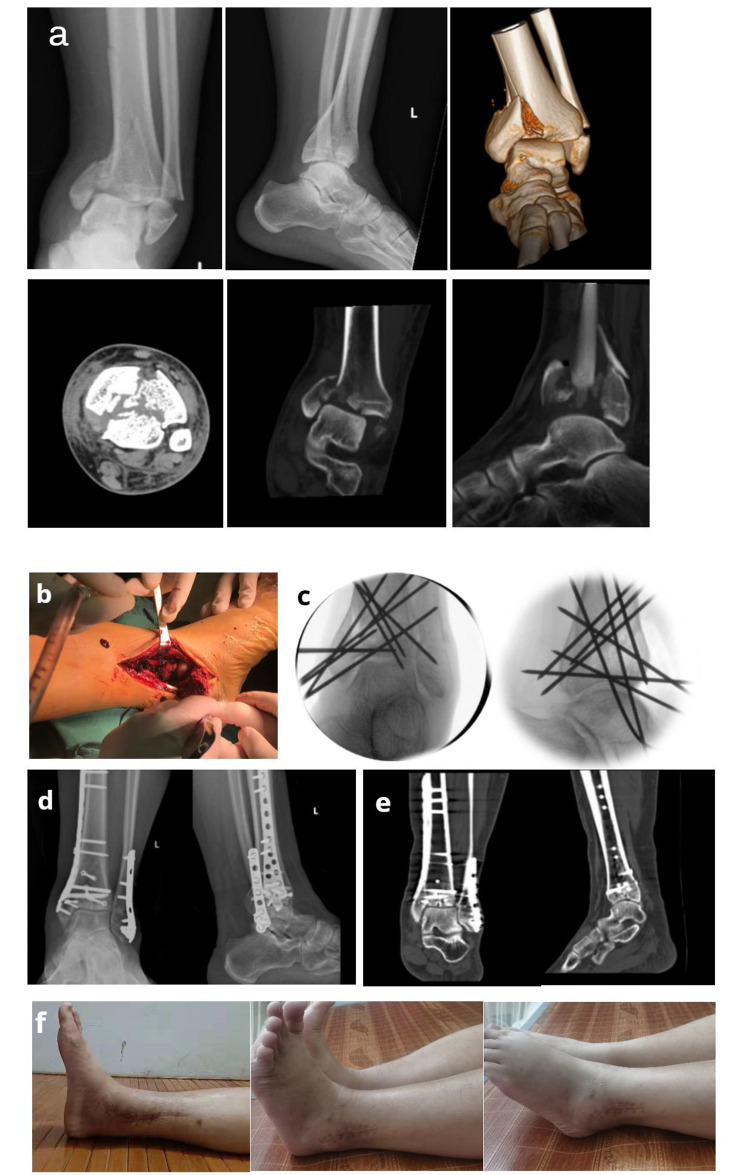
Photographs and radiographs of a 35-year-old male who experienced a varus-type tibial pilon fracture (AO/OTA type C3). (**a**) Preoperative X-ray radiographs and CT scans showed a varus-type pilon fracture characterized by compression of the medial column of the distal plafond. (**b**) Intraoperative medial malleolar window approach application (**c**) Intraoperative radiographs of K-wires temporary fixation technique. (**d ~ e**) Postoperative X-ray and CT examination showed a satisfactory reduction of the fracture. (**f**) Functional outcome

In the cases of varus-type tibial pilon fractures, the metaphyseal fractures were relatively simple accompanied by compression of the medial column of the distal plafond (Fig. 2a). Once the medial malleolar fragment had been inverted (Fig. 2b), we prioritized the reduction of the metaphyseal fragments, which could be temporarily fixed with Kirschner wires or cable, then we focused on reducing the distal intra-articular fractures. The lateral articular surface of the plafond was usually intact in varus-type tibial pilon fractures, and we used this intact articular surface as a reference to reduce other articular fracture segments. The posterior articular fragment was first reduced to the proximal tibial and the intact lateral articular segment with Kirschner wires temporarily placed from the proximal tibial into the posterior fragment or percutaneously from the lateral aspect of the distal tibial. Accurate reduction of the posterior fragment was critical as it provided a template for the subsequent reduction of the die-punch fractures and anterior fragments. Imprecise reduction of the posterior articular fragment could lead to joint incongruity and sagittal malalignment. After the posterior articular fractures had been reduced, Kirschner wire joysticks could be used to reduce the centrally impacted articular fragments. For this process, we needed to be particularly careful as the impacted fragments were usually comminuted. The articular surface reduction was adjusted repeatedly with reference to the normal dome shape of the distal tibial plafond. Once reduced satisfactorily, the central articular fragments could be secured with strategically-placed Kirschner wires (1.2 or 1.5 mm). Next, the anterior fragment was reduced to the distal tibial and fixed with temporary Kirschner wires. The accuracy of the reduction could be assessed by matching the extra-articular cortical read of the lateral segment and the articular read from the lateral aspect. The central osteochondral fragments could be further stabilized by running additional antero-posterior Kirschner wires through the anterior and posterior fragments. K-wires temporary fixation technique is shown in Fig. 2c. Autogenous or allogeneic bone graft transplants were needed when a cavity was present.

Once the reductions of the above fragments were complete, we evaluated the reductions by directly observing whether the articular surface was restored smoothly and the articular dome shape was recovered. The medial malleolar fragment was reversed superiorly to reduce to the remaining distal tibial followed by temporary fixation with Kirschner wires to match the extra-articular cortical read of the anterior segment. Finally, we checked the overall reduction with intraoperative C-arm fluoroscopy, and adjustments were made when necessary to ensure the integrity of the articular surface and restore the normal alignment of the ipsilateral lower limb.

**Fig. 3:**
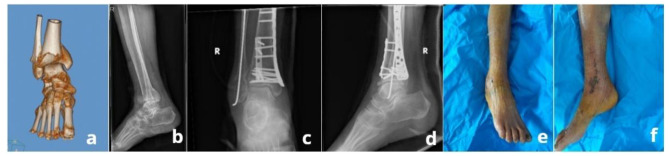
Photographs and radiographs from an 82-year-old female who experienced a varus-type pilon fracture (AO/OTA type C3). (**a ~ b**) Preoperative radiographs showed the fracture with a large anterior fragment involving the lateral column. (**c ~ d**) A 1/3 tubular plate was used to stabilize the anterior and posterior fragments, with the fibular fracture fixed intramedullary with a Kirschner wire. (**e ~ f**) The patient presented with local edge necrosis in the medial wound and the wound healed 12 days beyond the average healing time after dressing changes

For varus-type tibial pilon fractures, we typically used a low-profile medial buttress locking plate to complete the distal tibial fracture fixation (Fig. 2d) because it can effectively maintain the joint surface reduction and resist the varus deforming forces [20]. The medial plate should be placed carefully so that the distal screws can be fixed to the articular components as far as possible. It should first be secured distally to ensure that it fits the plafond, followed by fixation of the proximal screws. This process needs to be further confirmed by fluoroscopy. In situations of severe articular comminution or when fracture fragments were not adequately fixed with plate screws, additional fixation was required. Antero-posterior cortical screws or cannulated screws could be used for anterior and posterior fragment fixation simultaneously (Fig. 2d ~ e). For larger anterior fracture segments involving the lateral column of the distal plafond, a 1/3 tubular plate could also be used through the same medial approach and the posterior fragment could be stabilized by the distal long cortical screws through the tubular plate (Fig. 3a ~ d). After the distal tibial fracture was fixed, fixation of the fibula fracture was relatively simple. Usually, fibular fractures can be fixed intramedullary by closed reduction with Kirschner wires (Fig. 3c ~ d) or fixed with a plate through a lateral minimally invasive incision (Fig. 2d ~ f).

#### Anteromedial approach or extensile approach group

The anteromedial approach or extensile approach was selected according to the different fracture morphology of each patient. Usually, the anteromedial approach is fit for most varus-type pilon fractures to expose the medial column of the distal tibial fragments while the extensile approach is more suitable for cases when the anterior fracture fragment involves the lateral column of the distal tibial widely. A posterolateral approach was usually needed when displaced posterior fracture fragments occurred. Of course, MIPO technology could be selected according to the skin and soft tissue conditions of patients. Incision closure was performed after intraoperative fluoroscopic evaluation of fracture reduction and internal fixation.

### Postoperative plan

Patients were encouraged to perform toe activities and isometric muscle contraction soon after the surgery to avoid thrombosis. Early limb elevation was important for minimizing the tension of the skin closure. Active and passive ankle exercises were started three days after the surgery. The stitches were removed 2–3 weeks after surgery. Partial weight-bearing was initiated six weeks after surgery, and full weight-bearing functional exercise was started about 12 weeks after surgery depending on the status of fracture healing as shown by X-ray examination.

### Statistical analysis

SPSS23.0 (SPSS, USA) was used for all the data analysis. All the measurement data were checked by normal distribution first. An independent sample t-test was used for measurement data (age, follow-up period time, operation time, AOFAS) that fit the normal distribution, while those that didn’t meet were compared by the Mann-Whitney U test (days before surgery, hospitalization time, bone healing time, VAS). Fisher’s exact test was used for categorical variables (gender, injury side, complications, soft tissue classification, risk factor, clinical efficacy, and fracture reduction evaluation). All statistical analyses were considered significant when the p-value was < 0.05.

## Results

A total of 38 patients with pilon fractures were recruited for this study. The comparison results of the perioperative data between the two groups are shown in Table 1. The MMW approach group contained 9 males and 7 females with a mean age of 48.6 years (range: 34–82 years), while the conventional approach group included 15 males and 7 females with a mean age of 44.6 years (range: 29–62 years). All patients were followed up for over 12 months.

**Table 1:** Comparison of perioperative data between the MMW approach group and the Conventional approach group

Variables	MMW approach group(n = 16)	Conventional approach group(n = 22)	*P* value
Gender (n (%))			0.51
Male	9(56.25)	15(68.2)	
Female	7(43.75)	7 (31.8)	
Age (years, χ ± s)	48.6 ± 13.6	44.6 ± 9.0	1.07
Injured side (n (%))			1.0
Right	10(62.5)	14(63.6)	
Left	6 (37.5)	8 (36.4)	
Tscherne soft tissue classification (n (%))			1.0
0 1 2 3	2(12.5) 7(43.75) 6(37.5) 1(6.25)	3(13.6) 9(40.9) 9(40.9) 1(4.6)	
Risk factor (n (%))			0.93
Smoking	5(31.25)	8(36.4)	
Diabets	6(37.5)	12(54.5)	
Body mass index	7(43.75)	10(45.5)	
Injury to surgery (days)	6.5(6,7)	7(6,7)	0.9
Follow-up period (months, χ ± s)	15.8 ± 1.2	16.7 ± 1.2	2.4
Operation time (mins, χ ± s)	95.7 ± 4.6	118.8 ± 7.1	12.2
Hospitalization time (days)	12(12,13)	13(12,14)	2.0
Bone healing time (months)	13 (12,14)	13(13,14)	1.7
AOFAS at the last follow-up	91.4 ± 2.6	86.4 ± 3.2	5.2
VAS at the last follow-up	0 (0,0.75)	0(0,1)	0.7
Complications (n (%))	1(6.3)	1(4.5)	1.0

MMW = Medial Malleolar Window; AOFAS = American Orthopedic Foot and Ankle Society score; VAS = Visual Analogue Scale

From Table 1, we found that there was no significant difference between the two groups in gender, age, injured side, Tscherne soft tissue classification, prognostic risk factor, injury to operation time, hospitalization time, fracture healing time, or VAS. Interestingly, the MMW approach group has a shorter operation time with 95.7 ± 4.6 min compared with the conventional approach group with 118.8 ± 7.1 min. Meanwhile, the AOFAS is evidently higher in the MMW approach group at the last follow-up with 91.4 ± 2.6 points compared with the conventional approach group with 86.4 ± 3.2 points, even though the final statistical analysis showed that the above data are not statistically different.

The comparison results of clinical outcomes and fracture reduction assessment are shown in Table 2. The two groups differed significantly on the categorical variables in clinical effect and fracture reduction evaluation (p<0.05). The MMW approach group has excellent results in 13 cases and good in 3 cases, while excellent results were obtained in only 5 cases and good results were obtained in the remaining 17 cases in the conventional approach group. On the other hand, anatomical reductions were achieved in 15 cases and 1 case got a fair reduction for the MMW approach group, while 18 cases achieved an anatomical reduction and a fair reduction was obtained in the other 4 cases for the conventional approach group.

**Table 2:** Comparison of clinical outcomes and fracture reduction assessment

Variables	MMW approach group(n = 14)	Conventional approach group(n = 21)	*P* value
Clinical outcome by AOFAS (n (%))			0.001
Excellent	13(81.3)	5(22.7)	
Good	3(18.8)	17(77.3)	
Poor	0	0	
B-C radiographs criteria (n (%))			<0.001
Anatomical	15(93.75)	18(81.8)	
Fair	1(6.25)	4(18.2)	
Poor	0	0	

B-C radiographs criteria=Burwell-Charnley radiographs criteria

There was good wound healing at two weeks after surgery in all but two cases. One 82-year-old diabetic female in the MMW approach group, presented with a local wound edge necrosis, and the wound finally healed 12 days later than the average healing time after sterile dressing changes (Fig. 3e ~ f). Another case in the conventional approach group was treated with an anteromedial approach combining a posterolateral approach. Once the posterolateral incision had been sutured, the anteromedial approach could not be closed because of excessive tension, and vacuum-sealing drainage was used to cover the wound. The patient received a secondary closure of the anterolateral incision after the swelling subsided seven days later.

## Discussion

The choice of surgical incision is closely related to the pilon fracture pattern, and this opinion has become widely accepted by scholars [9, 21–23]. Pilon fractures can be classified into five different patterns according to the ankle position at the initial time of injury: valgus, varus, plantarflexion, dorsiflexion, and neutral. Many scholars [24–28] have investigated the morphology and characteristics of various types of pilon fractures. Determining the specific type of pilon fracture is critical for choosing the appropriate surgical approach and internal fixation strategies to facilitate reconstruction of the articular surface of the plafond and minimize postoperative complications. A detailed history of the patient’s ankle position at the time of injury and typical X-ray radiographs are of significance, also, CT scans play an important role in determining the type of fracture.

Extensive clinical studies have been conducted on common surgical approaches [9, 12, 19, 29, 30]. Each approach has unique characteristics and therefore, each approach is indicated for a different type of pilon fracture. The anteromedial or extensile anteromedial approach is often used to expose the medial column of the distal plafond in varus-type tibial pilon fractures, while the anterolateral approach is usually used to expose and reconstruct the lateral column easily in valgus-type tibial pilon fractures. The anterior or anteromedial approach is appropriate for dorsiflexion-type tibial pilon fractures, while the posterolateral or posteromedial approach can be used for plantarflexion-type tibial pilon fractures with posterior fragments. For complex pilon fractures involving multiple columns, a combination of approaches, such as anterior and posterior approaches, is widely used. Based on these traditional approaches, many scholars [11–14, 31, 33] have sought to develop new approaches for complex pilon fractures and satisfactory results have been reported. However, we also need to improve our understanding of the advantages and disadvantages of each approach and its respective indications.

The medial malleolar osteotomy technique through the standard medial approach has been applied for osteochondral lesions of the medial talar dome [16, 32]. The medial talar dome cartilage lesion can be exposed by the inverted osteotomized fragment, allowing for a direct view of the distal tibial articular surface at the same time. We believe that in pilon fractures with a large intact medial malleolar fragment, good visualization can also be obtained by inverting the medial malleolar fragment through the same standard medial approach. In this study, we initially selected cases of varus-type tibial pilon fractures using this medial malleolar window technique, such as those that are characterized by compression of the medial column of the distal plafond and often accompanied by a large medial malleolar fragment. After we released the soft tissue around the medial malleolar fragment and inverted the fragment, the distal tibial articular components were exposed. We could identify the exact position of each fracture fragment, and the distal tibial fractures could be reduced step-by-step with reference to the normal dome shape of the distal tibial plafond. The success of the final reduction was determined by whether the smoothness of the entire articular surface and the normal anterior distal tibial angle were restored. Satisfactory clinical results were obtained for all patients in the MMW approach group in our study, and the fracture reductions were also satisfactory according to the Burwell-Charnley radiograph criteria.

The medial malleolar window approach technique allowed for direct observation of the displaced fragments from the lateral aspect of the tibiotalar joint, as well as the direct assessment of the overall reduction of the articular surface. From this study, we can conclude that the biggest advantage of the medial malleolar window approach technique is the clear visualization of the whole distal tibial articular surface by inverting the medial malleolar fragment, and our study has also approved that all the cases in the MMW approach group got a satisfactory fracture reduction. Undoubtedly, this is a significant advantage over the previous surgical approaches. The anteromedial, anterolateral, and posterior approaches cannot achieve a direct visualization of the distal tibial articular surface [9]. Moreover, the reductions performed through these incisions may need to be evaluated by repeated intraoperative fluoroscopy, which poses potential harm to the doctors and patients and increases the operation time. In our cases, a universal distractor was not typically needed during the operation. Instead, a bump was placed under the ipsilateral ankle to move the ankle into a valgus position to obtain better visualization, which helped to reduce the operation time and risk of pin site infection.

Previous series have suggested that the anteromedial or extensile approaches are suitable for the reduction and fixation of the medial column of the distal tibial in varus-type tibial pilon fractures [9, 14, 15, 24]. For cases with displaced posterior articular fragments, a posterolateral or posteromedial approach is often needed for the exposure and stabilization of the posterior segment [24]. In our study, all cases in the conventional approach group were combined with a posterior incision. In contrast, a single medial malleolar window approach in our study can effectively reduce the posterior malleolus fragment under direct visualization and achieve stabilization of the posterior fragments through the same window using the antero-posterior screw technique or mini-plate fixation technique. In our study, the medial window approach has the advantages of shorter operation time and better functional recovery over the traditional double-incision approach. We believe that this is related to the fact that a single medial window approach can avoid another posterior incision, leading to reduced operation time and damage to the soft tissue. Importantly, the double incision technique still has related complications such as excessive suture tension, poor healing rate, and risk of deep infection [10]. It should be emphasized that the medial malleolar window approach technique is indicated when the varus-type pilon fracture has a considerably large medial malleolar fragment with the fracture line above the transverse distal tibial articular surface, ensuring the exposure of the entire articular surface after inverting the medial fragment. The medial malleolar window technique may not be appropriate for cases with a small or severely comminuted medial malleolus fracture.

This is a retrospective case-control study. Despite finding satisfactory clinical efficacy for the current report, further study in more cases is warranted, and randomized controlled trials are necessary to prove the advantages of this approach. Additionally, varus-type tibial pilon fracture injury patterns can be simulated in cadaveric specimens to verify the characteristics of this incision technique.

## Conclusion

In summary, the medial malleolar window approach provides excellent exposure to the distal tibial surface, allowing for satisfactory fracture reduction and functional rehabilitation. It is very suitable for varus-type tibial pilon fractures with a large intact medial malleolar fragment. We believe that this technique can greatly improve the surgical treatment of pilon fractures.

## Data Availability

The data of this study are available from the corresponding author upon reasonable request.
